# Antibody-Mediated Therapy in Gastric Cancer: Past, Present, and Future

**DOI:** 10.3390/cimb47121044

**Published:** 2025-12-15

**Authors:** Hong-Beum Kim, Sang-Gon Park

**Affiliations:** 1 Department of Premedical Course, Chosun University School of Medicine, 309 Pilmun-daero, Dong-gu, Gwangju 61452, Republic of Korea; redtiger@chosun.ac.kr; 2 Department of Internal Medicine, Hemato-Oncology, Chosun University Hospital, 365 Pilmun-daero, Dong-gu, Gwangju 61453, Republic of Korea

**Keywords:** gastric cancer, antibody-mediated therapy, HER2, VEGFR2, PD-1/PD-L1, CLDN18.2, immune checkpoint inhibitors, antibody–drug conjugates (ADCs), bispecific antibodies

## Abstract

The limited efficacy of cytotoxic chemotherapy in the context of gastric cancer treatment is largely driven by profound molecular and biological heterogeneity. In contrast, the development of antibody-mediated therapies has ushered in a new era of precision oncology by enabling selective molecular targeting and immune modulation. This review includes a comprehensive overview of the evolution of antibody-based therapeutics in gastric cancer, highlighting early breakthroughs, subsequent setbacks, and recent advances that have reshaped the treatment landscape. We summarize the current standard regimens targeting HER2, VEGFR2, PD-1/PD-L1, and CLDN18.2 and examine pivotal clinical trials evaluating monoclonal antibodies directed against these pathways. We also discuss emerging therapeutic modalities, including next-generation antibody–drug conjugates (ADCs), bispecific antibodies, and chimeric antigen receptor (CAR) T-cell therapies. Trastuzumab first established HER2-targeted therapy in gastric cancer, but the failure of trastuzumab emtansine (T-DM1) led to a decade-long stagnation until the advent of trastuzumab deruxtecan (T-DXd), which demonstrated robust clinical activity and defined a new standard of care. While bevacizumab failed to improve survival, the anti-VEGFR2 antibody ramucirumab emerged as an effective second-line therapy. Immune checkpoint inhibitors, including nivolumab and pembrolizumab, have been incorporated into first-line treatment for PD-L1-positive disease based on landmark trials such as CheckMate 649 and KEYNOTE-811. More recently, the CLDN18.2-targeted antibody zolbetuximab has expanded therapeutic options for biomarker-selected patients. Concurrently, a diverse pipeline of immune-based strategies—such as TROP2-directed ADCs, bispecific antibodies, and CAR-T cell therapies—is undergoing active clinical development. Together, advances in biomarker-driven antibody therapeutics are accelerating personalized cancer care and improving clinical outcomes in patients with gastric cancer.

## 1. Introduction

Until the early 2000s, systemic treatment for gastric cancer primarily relied on cytotoxic chemotherapy. However, due to the significant molecular heterogeneity and drug resistance of gastric cancer, chemotherapy alone struggled to achieve a median survival of over 12 months [1]. To overcome these limitations, advances in molecular biology, such as the TCGA project, shifted the treatment paradigm towards precision medicine. The identification of cancer cell surface antigens, in particular, led to the development of ‘antibody-mediated therapies’, which selectively attack cancer cells while protecting normal tissue [2]. In this review, we outline the evolution of antibody therapies for gastric cancer, tracing their history from established targets like HER2 and VEGF through the introduction of immune checkpoint inhibitors (PD-1/PD-L1) to the recently emerging CLDN18.2, and we discuss future prospects, including next-generation modalities.

## 2. Past and Present: Targets That Established the Standard for Gastric Cancer Antibody Therapy

**Figure 1:**
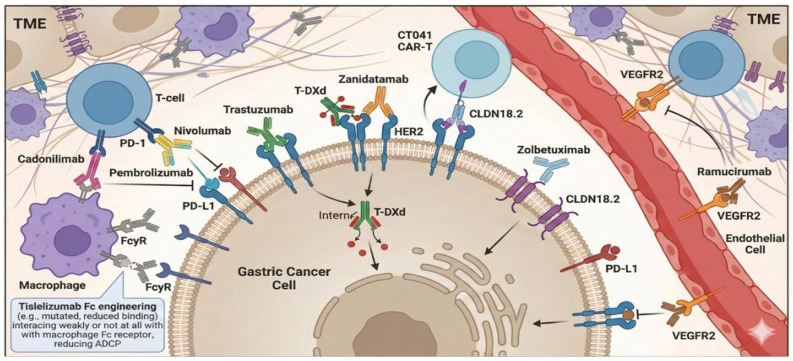
Overview of major molecular targets and mechanisms of action for antibody-based therapeutics within the gastric cancer microenvironment. This schematic provides an integrated overview of the principal antibody-based therapeutic strategies currently applied in gastric cancer, highlighting their targets and modes of action within the tumor microenvironment (TME). The figure illustrates representative approaches directed against tumor cells, immune components, and vascular endothelial cells, including HER2-targeted antibodies and antibody–drug conjugates, immune checkpoint inhibitors targeting the PD-1/PD-L1 axis, CLDN18.2-directed monoclonal antibodies, and VEGFR2 blockade to suppress tumor angiogenesis. Collectively, these strategies reflect the multi-compartmental nature of antibody-mediated therapy in gastric cancer and emphasize the interplay between tumor-intrinsic signaling, immune modulation, and microenvironmental regulation.

Antibody-based therapies have established a biomarker-driven treatment paradigm in gastric cancer by targeting cancer cell–associated antigens, immune checkpoints, and key pathways in the tumor microenvironment. The major therapeutic targets that formed the current standard of care include HER2, VEGF/VEGFR2, PD-1/PD-L1, and CLDN18.2. The key targets and mechanisms of action of antibody-based therapeutics in gastric cancer are summarized in Figure 1.

### 2.1. HER2

The human epidermal growth factor receptor 2 (HER2) is a key target that ushered in the era of antibody-mediated precision medicine in gastric cancer. HER2 was identified as a poor prognostic factor in breast cancer in the late 1980s, prompting research into its potential as a therapeutic target. In 1998, trastuzumab, a mAb-targeting HER2, was approved by the FDA, signaling the beginning of targeted anticancer therapy for solid tumors [3,4]. The exploration of HER2’s role in cancers other than breast cancer led to the discovery that HER2 overexpression or gene amplification was observed in approximately 15–20% of gastric cancer patients [5]. In 2010, the Trastuzumab for Gastric Cancer (ToGA) study identified HER2 as a key therapeutic target in gastric cancer, initiating the use of antibody-based anticancer therapy for this disease.

The ToGA study compared the effectiveness of combination therapy (standard chemotherapy with 5-fluorouracil and cisplatin plus trastuzumab) versus chemotherapy alone in patients with HER2-positive advanced or metastatic gastric or gastroesophageal junction adenocarcinoma. The trastuzumab combination therapy group demonstrated a statistically significant prolongation of median overall survival (mOS) of 13.8 months, compared to 11.1 months in the control group (HR = 0.74, *p* = 0.0046). Notably, combination therapy had a remarkable effect on the HER2 IHC 3+ subgroup, extending mOS up to 16 months. This study was the first to demonstrate an mOS exceeding 12 months in gastric cancer, rendering it significant as the first instance in which molecularly targeted therapy improved survival rates in gastric cancer [6].

Notwithstanding the success of the ToGA study, HER2 antibody-targeted therapy for gastric cancer has encountered numerous limitations over the past decade, failing to demonstrate the same dramatic efficacy seen in breast cancer. The primary reasons for this phenomenon included intratumoral heterogeneity, wherein HER2 expression levels exhibited significant variations within a single tumor, depending on its location. This heterogeneity was observed even in cases where the tumor was initially diagnosed as HER2-positive. Furthermore, treatment resistance emerged from the loss of gene amplification during therapy or the activation of alternative bypass pathways, such as EGFR or MET, which are other growth signaling pathways [5,6,7]. Subsequent studies were aimed at addressing these limitations; however, in contrast to the notable advancements observed in breast cancer research, the majority of efforts in gastric cancer research proved unsuccessful. For instance, lapatinib, a TKI that simultaneously inhibits HER2 and EGFR and had shown efficacy in breast cancer, failed to demonstrate improved overall survival when combined with chemotherapy in gastric cancer, as shown in the LOGIC study and the TyTAN study targeting Asian patients [8,9]. In the context of breast cancer, antibody–drug conjugates (ADCs) were explored as a novel form of antibody therapy. ADCs entail the targeted delivery of a cytotoxic anticancer drug connected to a monoclonal antibody via a linker to tumor cells, with the objective of eradicating cancerous cells [10,11]. In breast cancer, trastuzumab emtansine (T-DM1) has been established as a subsequent treatment modality following the success of the EMILIA study. However, in gastric cancer, the findings of the GATSBY study (mOS 7.9 months vs. 8.6 months; HR 1.15) similarly rendered HER2 therapy challenging [12]. The failure of T-DM1 in gastric cancer underscored key limitations of early-generation ADCs, including a relatively low drug-to-antibody ratio (DAR) and the lack of a bystander effect associated with a non-cleavable linker. This failure provided the impetus for researching new therapeutic approaches [7,12].

In 2017, early clinical data for trastuzumab deruxtecan (T-DXd), a completely novel ADC, was presented. T-DXd aimed to address the limitations of T-DM1. It combines a payload (topoisomerase I inhibitor) with a high DAR of up to 8, a linker cleavable selectively only within tumor cells, and potent cytotoxicity, while also possessing high cell membrane permeability to enable bystander killing effects capable of eliminating surrounding HER2-low or HER2-negative tumor cells [13]. T-DXd first demonstrated outstanding efficacy in breast cancer based on the 2019 DESTINY-breast results. Subsequently, in gastric cancer, the DESTINY-Gastric01 study involving patients in Japan and South Korea receiving third-line or later treatment showed significantly improved outcomes compared to standard chemotherapy in terms of objective response rate (ORR; 51% vs. 14%) and mOS (12.5 months vs. 8.4 months; HR = 0.59, *p* < 0.001). Consistent efficacy was, subsequently, replicated in the DESTINY-Gastric02 study involving Western patients [14,15].

In the recently published DESTINY-Gastric04 Phase 3 study comparing T-DXd with the standard second-line therapy ramucirumab plus paclitaxel in patients with HER2-positive gastric cancer who failed first-line therapy, the T-DXd group significantly improved mOS (14.7 months vs. 11.4 months; HR = 0.70, *p* = 0.0044) and progression-free survival (PFS) (6.7 months vs. 5.6 months) in the T-DXd group, demonstrating significant improvement. This advancement has elevated T-DXd to the status of a second-line standard treatment for HER2-positive gastric cancer [16]. Key clinical outcomes of major HER2-targeted clinical trials in gastric cancer are summarized in Table 1.

### 2.2. VEGF

Anti-angiogenesis has also emerged as a pivotal element within the field of oncology, with VEGF emerging as a particularly salient target. Antibody therapy targeting VEGF selectively binds to VEGF or its receptor (VEGFR), thereby blocking signaling that induces endothelial cell proliferation and migration, ultimately suppressing tumor angiogenesis at its source. This key mechanism has been demonstrated to impede the provision of nutrients and oxygen to tumors, thereby hindering the progression of cancer and the process of metastasis. Although BEVACIZUMAB, which has proven effective in the treatment of colorectal cancer, was studied in the context of gastric cancer, it did not demonstrate a survival benefit [17]. However, ramucirumab, a mAb targeting VEGFR-2, demonstrated efficacy in the RAINBOW and REGARD studies, confirming that the VEGF/VEGFR pathway is also a crucial therapeutic target in gastric cancer. It is currently employed as the prevailing second-line treatment for patients with gastric cancer [18,19]. The pivotal phase 3 clinical trials evaluating VEGF/VEGFR-targeted therapies in gastric cancer are summarized in Table 2.

### 2.3. PD-1/PD-L1: The Beginning of Immune Checkpoint Inhibitors

Immune checkpoint inhibitors (ICIs), frequently designated as third-generation cancer treatments, have been shown to reinvigorate the anti-tumor activity of suppressed T cells by obstructing immune checkpoint pathways such as PD-1/PD-L1. These pathways have been identified as a primary mechanism by which cancer cells evade the potent anti-tumor immune response of T cells, thereby demonstrating anticancer effects. However, the efficacy of ICIs is not uniform across all gastric cancers and is intimately linked to the tumor’s immunological profile. Gastric cancer can be broadly categorized into “hot” and “cold” tumors based on the TME. “Hot” tumors, represented primarily by Microsatellite Instability-High (MSI-H) and Epstein–Barr Virus (EBV)-positive subtypes, are characterized by a high tumor mutational burden (TMB) or the presence of viral neoantigens. These features promote robust T-cell infiltration, eliciting a strong endogenous immune response that makes these subtypes exceptionally responsive to ICIs. In contrast, “cold” tumors, such as the Genomically Stable (GS) or Chromosomal Instability (CIN) subtypes, often exhibit an immunosuppressive TME with limited T-cell infiltration, resulting in poor responses to ICI monotherapy. The molecular characterization of gastric cancer—including the identification of MSI-H and EBV-positive subtypes with strong immune activation—first demonstrated that specific GC subgroups may be intrinsically sensitive to immunotherapy [20,21]. Subsequently, the clinical success of PD-1 blockade in melanoma provided definitive proof of concept for ICI efficacy, which in turn catalyzed further investigation of immunotherapy in gastric cancer [22]. The ATTRACTION-2 Phase 3 trial was the inaugural study to demonstrate the potential of ICIs in gastric cancer, as it demonstrated that nivolumab monotherapy significantly prolonged mOS compared to placebo in patients with stage III or higher advanced gastric cancer (5.26 months vs. 4.14 months; Hr = 0.63, *p* < 0.0001) [23]. The KEYNOTE-061 study compared the efficacy of pembrolizumab monotherapy with that of paclitaxel in patients who had previously received first-line therapy, and the mOS did not differ significantly in the overall patient population. In the PD-L1 CPS ≥ 10 patient group, however, the HR of 0.64 suggests the potential for survival extension, thereby emphasizing the critical role of biomarker selection in ensuring the efficacy of ICI treatment [24].

Based on research on other cancer types, indicating that ICI may exhibit synergistic effects when combined with anticancer chemotherapy rather than as monotherapy, studies were also conducted on gastric cancer. The CheckMate 649 study was a Phase 3 trial that compared nivolumab plus chemotherapy (XELOX/FOLFOX) with chemotherapy alone as the first-line treatment in patients with HER2-negative advanced gastric cancer, gastroesophageal junction cancer, or esophageal adenocarcinoma. In the PD-L1 CPS ≥ 5 patient subgroup, nivolumab combination therapy demonstrated a significant improvement in mOS (14.4 months vs. 11.1 months: HR 0.71, *p* < 0.0001) and produced a cohort of patients who exhibited long-term survival. Based on these findings, the combination of nivolumab and chemotherapy has been established as the standard first-line treatment for patients with HER2-negative, PD-L1 CPS ≥ 5 advanced gastric cancer [25]. Similarly, the efficacy of pembrolizumab in combination with chemotherapy as a first-line treatment for HER2-negative advanced gastric cancer was investigated in the KEYNOTE-859 study. In the patient subgroup with a PD-L1 CPS ≥ 1, the pembrolizumab combination demonstrated a statistically significant improvement in median overall survival (HR 0.74), establishing it as another standard of care. A critical implication of this study is the expansion of the therapeutic landscape; unlike previous trials that primarily focused on high expressors, pembrolizumab demonstrated benefits across the broader CPS ≥ 1 population. This effectively extends the reach of ICI therapy to the PD-L1 low-expressing group (CPS 1–4), bridging the gap for patients who might otherwise fall outside the criteria for other ICI regimens [26].

The recently reported RATIONALE-305 study has expanded the therapeutic armamentarium by validating the efficacy of another PD-1 inhibitor, tislelizumab. Beyond confirming a survival benefit in the intention-to-treat population (HR 0.80), this study highlighted intriguing differentiators in subgroup analyses. Specifically, tislelizumab demonstrated consistent survival improvement trends in patients with peritoneal or liver metastases—subgroups where conventional ICIs have historically shown limited efficacy. This clinical distinction is mechanistically attributed to tislelizumab’s unique Fc-engineering, which minimizes binding to Fc receptors on macrophages, thereby reducing antibody consumption (antibody-dependent cellular phagocytosis) within the macrophage-rich tumor milieu. These findings suggest that future selection of ICIs should incorporate not only PD-L1 expression levels but also clinical characteristics such as metastatic sites and tumor burden, warranting further investigation into personalized immunotherapy strategies [27].

In parallel with these advancements in the HER2-negative setting, a triple combination therapy involving targeted therapy, immunotherapy, and chemotherapy has been attempted. The present study compared the efficacy of adding pembrolizumab to trastuzumab plus chemotherapy as a first-line treatment for patients with gastric cancer with that of a placebo group. In the initial interim analysis, the pembrolizumab combination group demonstrated an overwhelming response rate of 74.4%, in comparison to 51.9% in the placebo group, resulting in accelerated approval by the US FDA in 2021. The final analysis presented at the 2024 ESMO Congress demonstrated that following a median follow-up period of 50.2 months, the pembrolizumab combination group attained a mOS of 20.0 months. This outcome was statistically significant when compared to the 16.8 months observed in the placebo group (HR = 0.80, *p* = 0.004). This survival benefit was particularly marked in the PD-L1 CPS ≥ 1 patient subgroup, as evidenced by a significant difference in mOS (20.1 months vs. 15.7 months; HR = 0.79). These findings established triple combination therapy as the new standard of care for HER2-positive, PD-L1-positive gastric cancer [28]. Key phase 3 clinical trials investigating PD-1/PD-L1 inhibitors in advanced gastric cancer are summarized in Table 3.

### 2.4. CLDN18.2: Emergence of New Targets and Clinical Validation

Claudin proteins are pivotal membrane proteins that form tight junctions in normal epithelial cells. CLDN18.2 is expressed exclusively in gastric mucosal epithelial cells in normal tissue and remains hidden within the intercellular junction, thus avoiding exposure to the immune system. However, in the event of malignant transformation in gastric cancer, the loss of cellular polarity results in the exposure of CLDN18.2 on the surface of tumor cells. This characteristic, which is particularly evident in malignant tumors, has led to the proposal of a therapeutic target that can selectively attack cancer cells while minimizing damage to normal gastric tissue [29,30].

In early research, CLDN18.2 was found to be expressed in approximately 30–50% of all gastric cancer patients, with particularly high expression rates in the diffuse type and genetically stable (GS) subtypes. This finding indicated that it could serve as a new therapeutic target for patient groups that had previously experienced challenges from existing targeted therapies.

Zolbetuximab, a first-in-class IgG1 monoclonal antibody targeting CLDN18.2, mediates antitumor activity by inducing antibody-dependent cellular cytotoxicity (ADCC) and complement-dependent cytotoxicity (CDC) upon binding to CLDN18.2-expressing tumor cells [30]. The clinical feasibility of this approach was initially validated in a phase I dose-escalation study, which established a favorable safety profile for zolbetuximab monotherapy characterized by manageable gastrointestinal events and no maximum tolerated dose reached [31].

Building on this safety data, the randomized phase II FAST trial provided the first robust evidence of clinical efficacy. In this pivotal study, the addition of zolbetuximab to EOX chemotherapy significantly prolonged both progression-free survival (HR 0.44; *p* < 0.0005) and overall survival (HR 0.55; *p* < 0.0005) compared to chemotherapy alone in patients with CLDN18.2-positive disease. Notably, the survival benefit was most pronounced in patients with high CLDN18.2 expression (≥70% of tumor cells), validating CLDN18.2 as a predictive biomarker for patient selection [32]. These findings laid the foundation for recent global phase III trials, SPOTLIGHT and GLOW, positioning CLDN18.2-targeted therapy as a potential new standard of care.

The efficacy of zolbetuximab was confirmed in two large Phase 3 trials, SPOTLIGHT and GLOW, which evaluated the effectiveness of adding zolbetuximab to standard treatments FOLFOX or XELOX. Both studies included the enrollment of patients with HER2-negative, CLDN18.2-positive (moderate or strong membrane staining in ≥75% of tumor cells by IHC) advanced gastric cancer receiving first-line chemotherapy [33,34].

First, the SPOTLIGHT study included an evaluation of the combination effect of mFOLFOX6 chemotherapy, demonstrating that the zolbetuximab combination group significantly extended the median progression-free survival (PFS) compared to the placebo group: 10.61 months vs. 8.67 months (HR = 0.75, *p* = 0.0066). The mOS was also extended, from 18.23 months vs. 15.54 months (HR = 0.75, *p* = 0.0053), indicating a substantial increase [33]. Second, the GLOW study also evaluated the combination effect with CAPOX chemotherapy. In this study, the zolbetuximab combination group also showed a consistent improvement in median PFS compared to the placebo group: 8.21 months versus 6.80 months (HR = 0.687, *p* = 0.0007), and mOS of 14.39 months versus 12.16 months (HR = 0.771, *p* = 0.0118) [34].

The findings of the two studies provided compelling evidence that CLDN18.2 is the third key biomarker in first-line gastric cancer treatment, following HER2 and PD-L1. Zolbetuximab received approval in September 2024 from the European EMA and in October 2024 from the US FDA as a first-line treatment for HER2-negative, CLDN18.2-positive advanced gastric cancer. However, since CLDN18.2 is also expressed in normal gastric mucosa, gastrointestinal adverse events such as nausea and vomiting frequently occur. Therefore, proactive use of prophylactic antiemetics and patient education is essential for managing such events. Major phase 3 clinical trials of CLDN18.2-targeted therapy in advanced gastric cancer are summarized in Table 4.

## 3. Current Progress and Future Prospects in Antibody Therapy for Gastric Cancer

**Figure 2:**
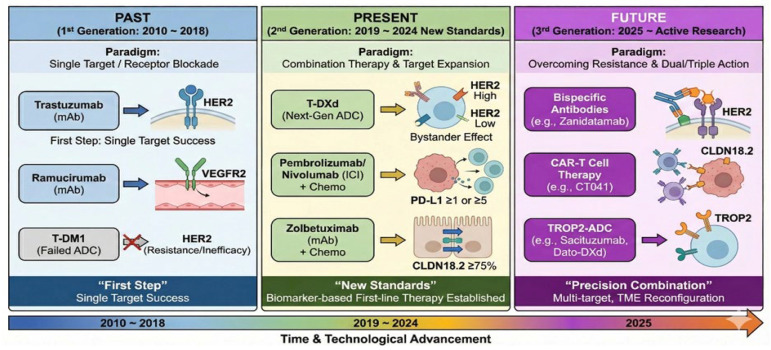
Evolution Schematic of Antibody Therapy in Gastric Cancer (Past, Present, Future). This diagram illustrates the progression of antibody-based therapeutic strategies across three distinct developmental phases, highlighting shifts in treatment paradigms. The Past (1st Generation: 2010–2018) panel depicts the initial “First Step” era focused on single-target receptor blockade, showing the success of monoclonal antibodies like trastuzumab targeting HER2 and ramucirumab targeting VEGFR2, alongside the limited efficacy of early ADCs like T-DM1 due to resistance mechanisms. The Present (2nd Generation: 2019–2024) panel outlines the establishment of "New Standards" driven by biomarker-based combination therapies and target expansion. Key advances include next-generation ADCs like T-DXd exhibiting a bystander effect across HER2 expression levels, immune checkpoint inhibitors (pembrolizumab/nivolumab) utilized based on PD-L1 status combined with chemotherapy, and zolbetuximab targeting high CLDN18.2 expression. The Future (3rd Generation: 2025 onward) panel demonstrates emerging research aimed at “Precision Combination” to overcome resistance and reconfigure the tumor microenvironment through dual/triple action modalities. These include bispecific antibodies (e.g., zanidatamab targeting HER2), CAR-T cell therapies directed at CLDN18.2 (e.g., CT041), and ADCs targeting novel antigens such as TROP2.Abbreviations: ADC, antibody-drug conjugate; CAR, chimeric antigen receptor; CLDN18.2, claudin 18.2; CPS, combined positive score; HER2, human epidermal growth factor receptor 2; ICI, immune checkpoint inhibitor; mAb, monoclonal antibody; PD-L1, programmed death-ligand 1; T-DM1, trastuzumab emtansine; T-DXd, trastuzumab deruxtecan; TME, tumor microenvironment; TROP2, trophoblast cell-surface antigen 2; VEGFR2, vascular endothelial growth factor receptor 2.

The therapeutic landscape of gastric cancer has undergone a paradigm shift with the introduction of antibody-based therapies targeting biomarkers such as HER2, PD-L1, and CLDN18.2. However, significant challenges persist, particularly the inevitable emergence of acquired resistance and the complex, immunosuppressive nature of the gastric tumor microenvironment (TME). The past, present, and future evolution of antibody-based therapy in gastric cancer is schematically illustrated in Figure 2. Consequently, current research efforts are increasingly focused on developing novel agents that exhibit precise binding affinity to these biomarkers while minimizing off-target toxicity. Key priorities for future development include elucidating strategies to overcome specific resistance mechanisms associated with each modality, identifying optimal combinations with chemotherapy, and breaching the immunological barriers of the TME. In this context, bemarituzumab, a first-in-class monoclonal antibody targeting FGFR2b, represents a promising advancement. In the phase 2 FIGHT study, bemarituzumab combined with mFOLFOX6 demonstrated a clinically meaningful improvement in progression-free survival (PFS) compared to placebo plus mFOLFOX6 (median PFS 9.5 vs. 7.4 months; hazard ratio [HR] 0.68) in patients with FGFR2b-overexpressing tumors [35]. These encouraging results have led to ongoing phase 3 validation. In the field of CLDN18.2-targeted therapy, the clinical success of zolbetuximab has catalyzed the development of next-generation antibodies designed to overcome resistance and improve efficacy. While zolbetuximab targets CLDN18.2, novel agents are being engineered for higher affinity and enhanced effector functions. Leading this wave is osemitamab (TST001), a high-affinity antibody with enhanced antibody-dependent cellular cytotoxicity (ADCC), designed to maintain efficacy even in patients with low-to-medium CLDN18.2 expression. In the TranStar 102 study (NCT04495296), a triple combination regimen comprising osemitamab, nivolumab, and CAPOX demonstrated encouraging results, achieving an objective response rate (ORR) of 76% and a median progression-free survival (mPFS) of 12.6 months [36]. Similarly, ASKB589 has emerged as a potent partner for immunotherapy. Results from a Phase Ib/II study (NCT05632939) presented at ASCO 2024 reported a confirmed ORR of 73.5% and a disease control rate (DCR) of 100% when combined with chemotherapy and a PD-1 inhibitor [37]. These findings provide a strong rationale for the “triple combination” strategy—integrating chemotherapy, immune checkpoint inhibitors, and CLDN18.2-targeted agents—to maximize clinical outcomes. Other novel agents are showing unique advantages in early-phase trials. LM-102 targets a unique epitope distinct from zolbetuximab to potentiate efficacy and has demonstrated excellent tolerability with no dose-limiting toxicity (DLT) up to 20 mg/kg (NCT04932628) [38]. ZL-1211 is biologically optimized to enhance tumor killing (NCT04263916) [39], while MIL93 utilizes afucosylation technology to maximize ADCC, showing manageable toxicity in early studies (NCT04665817) [40]. Furthermore, DR30303 distinguishes itself as a nanobody (VHH)-based fusion protein for superior tissue penetration (NCT05639153) [41]. In parallel, the evolution of antibody–drug conjugate (ADC) technology is fundamentally reshaping the treatment paradigm for gastric cancer. Unlike early-generation ADCs that predominantly utilized tubulin inhibitors and stable linkers, next-generation ADCs are engineered with cleavable linkers and potent cytotoxic payloads, such as topoisomerase I inhibitors. This structural optimization enables the “bystander antitumor effect,” allowing these agents to overcome tumor heterogeneity and demonstrate efficacy even in tumors with low target expression. For example, disitamab vedotin (RC48) targets HER2 but uses a different payload called monomethyl auristatin E (MMAE) attached to a different antibody site. This agent is of particular interest because it reportedly exhibits anticancer properties, even within the HER2 low-expression group. In early clinical studies, the drug achieved an objective response rate (ORR) of approximately 24% as a monotherapy. In a recently published Chinese primary treatment RCTS study, triple combination therapy with an ICI (tislelizumab) and an oral anticancer drug (S-1) demonstrated a high ORR of 92.1% and a median PFS of 12.6 months in patients with HER2-positive gastric cancer, raising expectations for further research [42,43].

Trophoblast cell-surface antigen 2 (TROP2) is a target whose expression is elevated in various cancers, including breast and lung cancer, and is associated with a poor prognosis. Sacituzumab Govitecan and Datopotamab Deruxtecan, which have already received approval for the treatment of breast cancer, are currently undergoing clinical trials for the treatment of gastric cancer [44,45]. Datopotamab deruxtecan employs the same DXd payload as T-DXd, and the results from the gastric cancer patient cohort in the TROPION-PanTumor01 Basket study are expected [46].

Bispecific antibody therapy involves a single antibody recognizing two different antigens simultaneously. This drug class can overcome tumor heterogeneity and maximize efficacy, and it has been extensively studied and applied in hematologic malignancies. Additionally, its use for solid tumors is being studied [47]. T-cell engagers, which simultaneously bind tumor cell surface antigens and immune cell activation receptors (primarily T cells), are being actively investigated in gastric cancer.

Givastomig is a representative drug that targets both CLDN18.2 and the T-cell co-stimulatory receptor 4-1BB, demonstrating anticancer effects by selectively amplifying T cells within the tumor microenvironment (TME). This drug demonstrated a very high ORR of 83% in a Phase 1 combination therapy study presented at ESMO GI 2025, proving its potential as a dual-specific antibody to become a significant future therapeutic mechanism in gastric cancer [48].

Following the logic of dual-targeting, ASP2138 represents another innovative approach within the CLDN18.2 landscape. Designed as a bispecific T-cell engager (BiTE) that simultaneously binds to CLDN18.2 on tumor cells and CD3 on T-cells, ASP2138 physically bridges T-cells to the tumor, triggering direct cytotoxicity. Early Phase 1 data have demonstrated a manageable safety profile and preliminary signals of antitumor activity in heavily pretreated patients, supporting the viability of T-cell engagement strategies beyond simple co-stimulation [49]. Expanding the horizon to HER2-positive gastric cancer, Zanidatamab (ZW25) has emerged as a transformative agent. Utilizing a biparatopic mechanism that binds simultaneously to two distinct domains (ECD4 and ECD2) of the HER2 receptor, it induces potent receptor clustering and downregulation. In the recent Phase 2b HERIZON-GEA-01 study, Zanidatamab combined with chemotherapy in the first-line setting achieved a remarkable objective response rate (ORR) of 84% and a disease control rate (DCR) of 93%, demonstrating superior efficacy compared to conventional monoclonal antibodies [50]. Furthermore, to dismantle the immunosuppressive barriers of the TME, cadonilimab (AK104) was developed as a first-in-class bispecific antibody concurrently targeting PD-1 and CTLA-4. In the pivotal Phase 3 COMPASSION-15 trial, cadonilimab combined with chemotherapy significantly prolonged overall survival compared to chemotherapy alone (median OS 15.0 vs. 10.8 months; HR 0.62) in the first-line treatment of advanced gastric cancer, thereby establishing a new benchmark for dual immune checkpoint blockade [51].

Targeting the Stromal Barrier and the “Bystander Effect”: One of the formidable challenges in gastric cancer therapeutics, particularly in diffuse-type or scirrhous gastric cancer, is the abundant desmoplastic stroma. This dense extracellular matrix acts as a physical barrier, hindering the uniform distribution of high-molecular-weight antibody drugs [52]. To address this, the concept of Cancer Stromal Targeting (CAST) therapy has been proposed. This strategy utilizes cytotoxic immunoconjugates that bind to stromal targets, releasing their payload to destroy both the stromal barrier and adjacent tumor cells. Recent evidence suggests that the superior efficacy of T-DXd in HER2-low tumors is partly attributable to this mechanism; unlike conventional ADCs, the payload of T-DXd is released extracellularly after cleavage, exerting a bystander effect not only on neighboring tumor cells but potentially on the stromal components as well [53]. Furthermore, novel agents specifically designed to target the stroma are under active investigation. For instance, PYX-201, an ADC targeting the extradomain B (EDB) of fibronectin—an oncofetal antigen abundant in the tumor stroma—has recently received FDA Fast Track designation based on promising Phase 1 data, highlighting the potential of stroma-directed strategies to overcome the limitations of the gastric TME [54].

While antibody-based modalities continue to expand the therapeutic landscape, chimeric antigen receptor T-cell (CAR-T) therapy has emerged as a breakthrough strategy that reprograms autologous T cells to directly recognize and eliminate tumor antigens. After leukapheresis, patient T cells are genetically engineered to express a CAR targeting a selected tumor-associated antigen and reinfused to mediate targeted cytotoxicity. CAR-T therapy has shown remarkable clinical activity in hematologic malignancies, establishing it as a transformative immunotherapeutic platform. However, translating this success to solid tumors—including gastric cancer—remains challenging due to multiple barriers, such as limited intratumoral trafficking and infiltration, antigen heterogeneity leading to escape, and a profoundly immunosuppressive tumor microenvironment that drives T-cell dysfunction and exhaustion [55].

In the context of gastric cancer, Satricabtagene autoleucel (CT041), an autologous CAR-T cell therapy targeting CLDN18.2, has demonstrated the most advanced and promising clinical data to date. According to interim results from a pivotal Phase 1 trial (NCT03874897) published in *Nature Medicine*, CT041 exhibited a manageable safety profile and significant efficacy in heavily pretreated patients with gastrointestinal cancers. Specifically, within the gastric cancer subgroup—comprising patients who had progressed after at least two prior lines of systemic therapy—CT041 achieved an impressive objective response rate (ORR) of 57.1% and a 6-month overall survival rate of 81.2%. These findings serve as a critical proof-of-concept that engineered CAR-T cells can successfully penetrate the desmoplastic tumor microenvironment of gastric adenocarcinoma and induce meaningful tumor regression, paving the way for ongoing confirmatory Phase 2 studies [56]. While CLDN18.2 remains the frontrunner, the inherent heterogeneity of gastric cancer necessitates a broader spectrum of therapeutic targets to mitigate the risk of antigen escape. Consequently, early-phase clinical trials are actively exploring CAR-T cells directed against alternative tumor-associated antigens (TAAs), including EGFR, EpCAM, and MUC1.EGFR-targeted CAR-T cells are currently under investigation in advanced solid tumors, including gastric cancer, aiming to exploit the frequent EGFR overexpression observed in a subset of patients [57]. Similarly, EpCAM, a widespread marker on epithelial carcinomas, and MUC1, a glycoprotein aberrantly glycosylated in gastric tumors, serve as attractive targets for next-generation CAR-T constructs [58,59]. Although these studies are predominantly in the dose-escalation phase, focusing on safety and feasibility, they represent a critical step toward diversifying the therapeutic armamentarium and enabling precision cellular immunotherapy tailored to individual antigen profiles.

The most significant challenge in antibody-based therapy is the emergence of treatment resistance. For gastric cancer, the predominant mechanisms that confer resistance to HER2-targeted therapy have been identified as the loss of HER2 amplification after treatment and the activation of alternative pathways, such as MET and EGFR [5,7,60]. Even potent ADCs like T-DXd are subject to resistance mechanisms, including reduced HER2 expression, activation of drug efflux pumps, and enrichment of immunosuppressive cells within the TME. Additional mechanisms involve defects in the IFN-γ signaling pathway and JAK/STAT mutations [61].

Despite ongoing research aimed at overcoming these challenges, the extensive array of resistance mechanisms poses significant obstacles to the development of the next generation of therapeutic agents. Concurrently, research endeavors are underway to develop real-time diagnostic methods capable of detecting HER2 amplification loss or the emergence of new resistance mutations in circulating tumor DNA (ctDNA) through frequent analysis. Once resistance is confirmed, the objective is to promptly transition to the subsequent treatment strategy (e.g., switching to T-DXd or multi-target combination therapy) to overcome resistance [1]. As rapid progress is made, determining which drug to use first and the sequence of drug administration when multiple biomarkers for gastric cancer antibody therapy are simultaneously positive also remains a challenge to be addressed.

Crucially, in the current therapeutic landscape, the assessment of Microsatellite Instability-High (MSI-H) or Deficient Mismatch Repair (dMMR) and Epstein–Barr Virus (EBV) status must constitute the first step in the treatment decision algorithm, taking precedence over other biomarker considerations.

Patients with MSI-H/dMMR or EBV-positive tumors exhibit a distinct “hot” immunological profile characterized by robust T-cell infiltration and high immunogenicity. Consequently, these subtypes demonstrate exceptional sensitivity to immune checkpoint inhibitors (ICIs). For MSI-H patients, ICI-based regimens should be prioritized as the absolute standard of care, often yielding durable long-term survival that surpasses the benefits of other targeted therapies. Therefore, the complex sequencing of HER2, PD-L1, and CLDN18.2 targeted agents is primarily relevant for the majority of patients who are Microsatellite Stable (MSS) [2,20,62]. For these MSS patients, determining priority among the three independent biomarker-based therapies—HER2, PD-L1 (CPS), and CLDN18.2—remains a paucity of research. The reason for this is that clinical studies often establish distinct, treatment-specific target patient populations, and there is also a lack of head-to-head comparative studies [1].

Clinicians must select the optimal treatment through reasonable inference based on indirect evidence. A subsequent discussion will encompass several such cases. For patients with HER2-positive and PD-L1-positive (CPS ≥ 1) diagnoses, the combination therapy of chemotherapy, trastuzumab, and pembrolizumab, as demonstrated in the KEYNOTE-811 study, offers the strongest evidence supporting its use. This therapeutic approach has yielded a noteworthy mOS of 20 months, marking the most protracted survival period documented thus far in first-line treatment for gastric cancer. Therefore, regardless of CLDN18.2 expression status, it is reasonable to prioritize this triple therapy regimen [28]. However, a distinction must be made for the subset of HER2-positive patients with negative PD-L1 expression (CPS < 1). Updated subgroup analyses from the KEYNOTE-811 trial indicated that the addition of pembrolizumab failed to demonstrate a survival benefit in this population, with hazard ratios for overall survival crossing unity. Consequently, regulatory bodies, including the FDA, have revised approvals to restrict the triple regimen to PD-L1-positive cases. For HER2-positive but PD-L1-negative patients, the conventional doublet regimen of trastuzumab plus chemotherapy remains the gold standard.

In the scenario where a patient is HER2-negative and exhibits low CLDN18.2 expression but is PD-L1 positive, the therapeutic strategy should fundamentally anchor on ICI-based combinations. However, the specific choice of agent may be nuanced by the magnitude of PD-L1 expression. For patients with a CPS ≥ 5, the addition of nivolumab to chemotherapy should be considered the primary option, supported by the robust survival benefits and established regulatory approvals derived from the CheckMate 649 trial [25]. Conversely, for patients falling into the lower expression range—specifically those with a CPS 1–4 subgroup—who may not meet the strict reimbursement or approval criteria for nivolumab in certain jurisdictions, pembrolizumab plus chemotherapy presents a viable therapeutic alternative. This approach is substantiated by the KEYNOTE-859 study, which demonstrated efficacy in the broader CPS ≥ 1 population, thereby bridging the therapeutic gap for patients with low-positive PD-L1 expression [26]. Specifically, in the HER2-negative and PD-L1-negative setting, zolbetuximab plus chemotherapy has emerged as the standard of care for CLDN18.2-positive patients, underscoring the necessity of testing for this biomarker to ensure no therapeutic opportunity is missed [33,34].

Subsequently, a significant therapeutic overlap exists for patients with HER2-negative status who are co-positive for PD-L1 and CLDN18.2. In this scenario, clinicians must weigh immune checkpoint inhibitor (ICI)-based regimens—comprising either nivolumab (based on CheckMate 649, typically for CPS ≥ 5) or pembrolizumab (based on KEYNOTE-859, approved for CPS ≥ 1)—against the CLDN18.2-targeted regimen of zolbetuximab plus chemotherapy (based on SPOTLIGHT/GLOW). While all these regimens have demonstrated superior survival benefits compared to chemotherapy alone, there is an absence of direct head-to-head comparison data. The selection of treatment should be individualized based on the patient’s comorbidities, the anticipated toxicity profile, and treatment goals. Those prioritizing the potential for long-term survival—a hallmark “tail-of-the-curve” benefit associated with ICIs—may opt for ICI-based therapy (nivolumab or pembrolizumab). On the other hand, those seeking to impede the onset of disease progression, by virtue of zolbetuximab’s documented efficacy in significantly enhancing progression-free survival (PFS), may advocate for CLDN18.2-targeted therapy. The necessity of a prospective randomized comparative clinical trial targeting patients positive for multiple biomarkers is hereby proposed.

## 4. Conclusions

Antibody-mediated therapy for gastric cancer has undergone significant advancements over the past two decades, marking the advent of a new era in precision medicine that utilizes molecular targets and the immune system, shifting from reliance on cytotoxic chemotherapy. The advent of trastuzumab, a drug that targets HER2, has led to the prospect of utilizing biomarker-based therapy in the treatment of gastric cancer. Innovative ADCs, such as T-DXd, which surmounted their limitations, advanced the treatment paradigm to the next level. ICIs have fundamentally reshaped the landscape of first-line therapy through the PD-L1 biomarker, and the recent success of CLDN18.2-targeted therapy is raising expectations for new treatment modalities.

However, despite significant progress, the road to conquering stomach cancer remains arduous and lengthy. Intrinsic and acquired resistance originating from tumor heterogeneity persists as a foundational challenge for all antibody therapies. Severe toxicities, such as ILD and irAEs, act as factors impeding treatment. Furthermore, the selection of optimal treatment for patients who are positive for multiple biomarkers, as well as the development of rational sequential treatment strategies following the failure of new standard therapies, represent unmet needs that require further research.

The pivotal element for the advancement of gastric cancer antibody therapy is a “personalized combination and sequencing” strategy that is meticulously designed to circumvent existing challenges. This will be realized by transitioning from single-target, single-drug methodologies to more comprehensive approaches, such as ▲ the introduction of next-generation platform technologies, such as bispecific antibodies and CAR-T; ▲ real-time resistance monitoring via liquid biopsy; ▲ deep understanding of the tumor microenvironment using advanced technologies like spatial transcriptomics; and ▲ the discovery of novel regulatory factors, such as the gut microbiome.

In conclusion, the field of gastric cancer antibody therapy has evolved in a manner that builds upon prior achievements, thereby establishing contemporary standards and overcoming future limitations through progressively more sophisticated and multifaceted approaches. The fundamental mission of oncologists is to translate these scientific advances into improved survival rates and enhanced quality of life for actual patients through close collaboration between clinical and translational research. This will be an important milestone on the path toward transforming gastric cancer from an incurable disease into a manageable chronic condition.

## Figures and Tables

**Table 1:** Summary of HER2-targeted clinical trials in gastric cancer.

	Line/Setting	Regimen	ORR (%)	mPFS (mo)	mOS (mo)	Study
Trastuzumab	1L	FP (orXP) + Cisplatin + Trastuzumab vs. FP (or XP) + Cisplatin	47 vs. 35	6.7 vs. 5.5 (HR = 0.71)	13.8 vs. 11.1 (HR = 0.74)	ToGA
Lapatinib	1L	Lapatinib + CapeOx vs. Placebo + CapeOx	53 vs. 39	6.0 vs. 5.4 (HR = 0.82) *	12.2 vs. 10.5 (NS)	LOGiC (TRIO-013)
Lapatinib	2L (Asian)	Lapatinib + Paclitaxel vs. Paclitaxel	27 vs. 9	5.4 vs. 4.4	11.0 vs. 8.9 (NS)	TyTAN
Trastuzumab emtansine (T-DM1)	2L	T-DM1 vs. Taxane	20.6 vs. 19.6	2.7 vs. 2.9	7.9 vs. 8.6 (HR = 1.15)	GATSBY
Trastuzumab deruxtecan (T-DXd)	3L+ (JP/KR)	Trastuzumab deruxtecan (T-DXd) vs. Chemo (physician’s choice)	51 vs. 14	5.6 vs. 3.5 (HR = 0.47)	12.5 vs. 8.4 (HR = 0.59)	DESTINY-Gastric01
Trastuzumab deruxtecan (T-DXd)	2L (Western)	T-DXd (single-arm)	41.8	5.6	12.1	DESTINY-Gastric02
Trastuzumab deruxtecan (T-DXd)	2L	T-DXd vs. Ramucirumab + Paclitaxel	44.3 vs. 29.1	6.7 vs. 5.6	14.7 vs. 11.4 (HR = 0.70)	DESTINY-Gastric04

Abbreviations: 1L, first line; 2L, second line; ORR, objective response rate; mPFS, median progression-free survival; mOS, median overall survival; HR, hazard ratio; NS, not significant; FP, 5-fluorouracil plus cisplatin; XP, capecitabine plus cisplatin; Chemo, chemotherapy; JP, Japan; KR, Korea. * No statistically significant difference was observed between the treatment arms.

**Table 2:** Key phase 3 trials of VEGF/VEGFR-targeted therapy in gastric cancer.

	Line/Setting	Regimen	ORR (%)	mPFS (mo)	mOS (mo)	Study
Bevacizumab	1L	Bevacizumab + capecitabine + cisplatin vs. Placebo + capecitabine + cisplatin	46.0 vs. 37.4	6.7 vs. 5.3 (HR = 0.80, *p* = 0.0037)	12.1 vs. 10.1 (HR = 0.87, *p* = 0.10)	AVAGAST
Ramucirumab	2L	Ramucirumab vs. Placebo	3.4 vs. 2.6 (NS)	2.1 vs. 1.3 (HR = 0.48, *p* < 0.0001)	5.2 vs. 3.8 (HR = 0.78, *p* = 0.047)	REGARD
Ramucirumab	2L	Ramucirumab + Paclitaxel vs. Placebo + Paclitaxel	28.0 vs. 16.0	4.4 vs. 2.9 (HR = 0.64, *p* < 0.0001)	9.6 vs. 7.4 (HR = 0.81, *p* = 0.017)	RAINBOW

Abbreviations: 1L, first line; 2L, second line; ORR, objective response rate; mPFS, median progression-free survival; mOS, median overall survival; HR, hazard ratio; NS, not significant; Cape, capecitabine; Cis, cisplatin.

**Table 3:** Key clinical trials of PD-1/PD-L1 inhibitors in advanced gastric cancer.

	Line/Setting	Regimen	ORR (%)	mPFS (mo)	mOS (mo)	Study
Nivolumab	3L, Asia, All-comers	Nivolumab vs. Placebo (PD-L1 not required);	11.2 vs. 0.0	1.61 vs. 1.45 (HR 0.60)	5.26 vs. 4.14 (HR 0.63; *p* < 0.0001)	ATTRATION-2
Pembrolizumab	2L, HER2(−) G/GEJ	Pembrolizumab vs. Paclitaxel	16.3 vs. 13.6	1.5 vs. 5.4 (HR 0.82) *	9.1 vs. 8.3 (HR 0.82 *p* = 0.042; not significant)	KEYNOTE-061
Nivolumab	1L, HER2(−) G/GEJ/esophageal adenocarcinoma; CPS 5	Nivolumab + Chemo (FOLFOX/XELOX) vs. Chemo	60 vs. 45 (3-y update)	7.7 vs. 6.0 (HR 0.68)	14.4 vs. 11.1 (HR 0.71; *p* < 0.0001)	CheckMate 649
Pembrolizumab	1L, HER2(−) G/GEJ/esophageal adenocarcinoma; CPS ≥ 1	Pembrolizumab + chemo FP (5-FU + cisplatin or CAPOX vs. chemo	52.1 vs. 43.0	6.9 vs. 5.6	13.0 vs. 11.4 (HR 0.74)	KEYNOTE-859
Pembrolizumab	1L, HER2(+) metastatic G/GEJ; global	Pembrolizumab + Trastuzumab + Chemo vs. Placebo + Trastuzumab + Chemo	74.6 vs. 60.1 (interim)	10.0 vs. 8.1 (HR ~0.73) CPS ≥ 1: 10.9 (Exp) vs. 7.3 (Ctrl) (HR ≈ 0.71	20.0 vs. 16.8 (HR 0.80; *p* = 0.004); CPS ≥1: 20.1 vs. 15.7 (HR 0.79)	KEYNOTE-811
Tislelizumab	1L, PD-L1 All-comers	Tislelizumab 200 mg Q3W + investigator-chosen (oxaliplatin + capecitabine or cisplatin + 5-FU) vs. placebo + chemo	47.3 vs. 40.5	6.9 vs. 6.2 HR 0.78	15.2 vs. 12.9 (HR 0.80; *p* = 0.001)	RATIONALE-305

Abbreviations: 1L, first line; 2L, second line; 3L, third line; ORR, objective response rate; mPFS, median progression-free survival; mOS, median overall survival; HR, hazard ratio; NS, not significant; G/GEJ, gastric/gastroesophageal junction; CPS, combined positive score; Chemo, chemotherapy; Exp, experimental arm; Ctrl, control arm. * The difference did not reach statistical significance.

**Table 4:** Phase 3 trials of CLDN18.2-targeted therapy in advanced gastric cancer.

	Line/Setting	Regimen	ORR (%)	mPFS (mo)	mOS (mo)	Study
zolbetuximab	Phase 3/1L CLDN18.2+, HER2(−) (global)	Zolbetuximab + mFOLFOX6 vs. Placebo + mFOLFOX6 (CLDN18.2 ≥ 75%)	No meaningful difference (48% both arms) *	10.61 vs. 8.67 (HR = 0.751; *p* = 0.0066)	18.23 vs. 15.54 (HR = 0.75; *p* = 0.0053)	SPOTLIGHT
zolbetuximab	Phase 3/1L CLDN18.2+, HER2(−) (global)	Zolbetuximab + CAPOX vs. Placebo + CAPOX (CLDN18.2 ≥ 75%)	No difference (42.5% vs. 40.3) both arms) *	8.21 vs. 6.80 (HR = 0.687; *p* = 0.0007)	14.39 vs. 12.16 (HR = 0.771; *p* = 0.0118)	GLOW

Abbreviations: 1L, first line; ORR, objective response rate; mPFS, median progression-free survival; mOS, median overall survival; HR, hazard ratio; CLDN18.2+, claudin 18.2–positive; mFOLFOX6, modified FOLFOX6 (oxaliplatin, leucovorin, and fluorouracil); CAPOX, capecitabine plus oxaliplatin. * ORR was similar between treatment arms.

## Data Availability

No new data were created or analyzed in this study. Data sharing is not applicable to this article.
